# Sublingual immunotherapy tablet: a cost-minimizing alternative in the treatment of tree pollen-induced seasonal allergic rhinitis in Canada

**DOI:** 10.1186/s13223-021-00565-y

**Published:** 2021-07-08

**Authors:** Anne K. Ellis, Rémi Gagnon, Eva Hammerby, Julia Shen, Sheena Gosain

**Affiliations:** 1 Division of Allergy and Immunology, Department of Medicine, Queen’s University, Kingston, ON Canada; 2 Service D’Allergie Et Immunologie, Département de Médecine, CHU de Québec, Québec City, QC Canada; 3 ALK-Abello A/S, Hørsholm, Denmark; 4 PDCI Market Access Inc., Ottawa, ON Canada

**Keywords:** Cost-minimization, Tree pollen, Birch pollen, Sublingual immunotherapy, Allergy immunotherapy, Allergic rhinitis

## Abstract

**Background:**

A cost-minimization analysis (CMA) was performed to evaluate the economic implications of introducing the SQ Tree sublingual immunotherapy (SLIT)-tablets marketed as ITULATEK® (Health Canada regulatory approval in April 2020) for the treatment of pollen-induced (birch, alder and/or hazel) seasonal allergic rhinitis in Canada (Ontario and Quebec), where Tree Pollen subcutaneous immunotherapy (SCIT) is already an available treatment option.

**Methods:**

A CMA was deemed appropriate and was based on the assumption that the SQ Tree SLIT-tablets have comparable efficacy to Tree Pollen SCIT. A societal perspective was adopted in the model, including relevant costs of medications, costs of health care services, and productivity losses. The time horizon in the model was three years, which corresponds to a minimal treatment course of allergy immunotherapy. Resource use and costs were based on published sources, where available, and validated by Canadian specialist clinicians (allergists) in active practice in Ontario and in Quebec, where applicable. A discount rate of 1.5% was applied in accordance with the Canadian Agency for Drugs and Technologies in Health (CADTH) guidelines. To assess the robustness of the results, scenario analyses were performed by testing alternative assumptions for selected parameters (e.g., Tree Pollen SCIT resource use, discount rates, number of injections, annual SCIT dosing with maintenance injections, and nurse time support), to evaluate their impact on the results of the analysis.

**Results:**

The direct costs, including the drug costs, and physician services costs, for three years of treatment, were similar for both SQ Tree SLIT-tablets vs. Tree Pollen SCIT in both Ontario and Quebec ($2799.01 and $2838.70 vs. $2233.76 and $2266.05 respectively). However, when the indirect costs (including patient’s travel expenses and lost working hours) are included in the model, total savings for the treatment with SQ Tree SLIT-tablets of $1111.79 for Ontario and $1199.87 for Quebec were observed. Scenario analyses were conducted and showed that changes in assumptions continue to result in the savings of SQ Tree SLIT- tablets over Tree Pollen SCIT.

**Conclusions:**

The CMA indicates that SQ Tree SLIT-tablets are a cost-minimizing alternative to Tree Pollen SCIT when considered from a societal perspective in Ontario and Quebec.

## Background

Allergic rhinitis (AR) affects individuals worldwide with increasing prevalence. In Canada, AR is estimated to impact about 20 to 25% of the population, and more than half of these people are not well controlled on conventional medications [1]. Individuals suffer from a high symptom burden and associated consequences, which can have a considerable negative impact on patient productivity and quality of life, resulting in a substantial economic burden.

Tree pollen is one of the most common inhalant allergens that can cause AR, and birch pollen is the major tree pollen allergen across most of Canada [2]. The prevalence of allergic sensitization (skin test greater than 3 mm to any allergen) in sensitized patients who presented themselves with suspected allergy has been shown in the province of Saskatchewan to be as high as 32.1% for mixed grass and 26.8% for birch [3]. In Edmonton, Alberta, the prevalence of positive skin test to grasses and birch in sensitized patients has been shown to be 39.2 and 23.7%, respectively [4]. The prevalence of atopic sensitization from 14 allergens in adults 20 to 44 years in six study sites across Canada (Vancouver, Winnipeg, Hamilton, Montreal, Halifax, and Prince Edward Island) found that, on average, the proportion of patients sensitized to birch pollen was 15.2% (95% CI 14.3–17.1) [5]. Individuals with birch pollen induced AR experience symptoms which may last several months each year due to the cross-reactivity of birch and related species. Immunological cross-reactivity between pollens from the birch homologous group (alder, hornbeam, hazel, oak, and beech) leads to individuals sensitized to birch pollen to also experience symptoms when exposed to pollen from related species [6, 7]. There is significant variation in pollen counts and season length across Canada due to geographic location and environmental factors which can change year to year based on climate [3, 5, 8]. Pollen data from 2016 to 2018 reported that the birch pollen season in Ontario and Quebec could last up to 7 weeks [8].

Several treatment options aimed to reduce the symptoms related to allergic rhinitis are available, including allergen avoidance, oral and intranasal antihistamines, intranasal corticosteroids, combination intranasal corticosteroid/antihistamine sprays, and leukotriene receptor antagonists (LTRAs). For patients with persistent AR despite the use of pharmacologic therapies and evidence of specific IgE antibodies to clinically relevant allergens, allergy immunotherapy (AIT) is indicated [9–11]. AIT is either administered as subcutaneous immunotherapy (SCIT) via injections or as sublingual immunotherapy (SLIT) via tablets, both of which are approved in Canada. A typical treatment duration for AIT is 3–5 years; studies of AIT demonstrated that 3 years of continuous treatment with SCIT or SLIT produces a prolonged remission of symptoms [12].

The SQ Tree SLIT-tablets containing 12 SQ-Bet (a measure of the biological allergen activity based equally on the major allergen content (Bet v 1) and total allergenic activity) of standardized natural birch pollen extract of White Birch (*Betula verrucosa*) (ITULATEK®) are approved by Health Canada as an allergy immunotherapy for the treatment of moderate to severe seasonal allergic rhinitis, with or without conjunctivitis, induced by pollen from birch, alder and/or hazel [13]. The treatment is indicated in adults 18–65 years of age who have a clinical history of symptoms of AR, despite use of symptom-relieving medication, and a positive test of sensitization to one or more of the pollen of birch, alder or hazel (skin prick test and/or specific IgE).

The cost-minimization analysis (CMA) described here was performed to evaluate the economic implications of introducing SQ Tree SLIT-tablets in Canada, where Tree Pollen SCIT is already available as a treatment option.

## Methods

### Cost minimization analysis

The CMA was used to estimate the economic impact of SQ Tree SLIT-tablets (*B. verrucosa*, 12 SQ-Bet, ALK, Denmark; available across Canada) compared with other AIT options, i.e. SCIT, available in Canada for the treatment of AR (with or without conjunctivitis) induced by birch tree, alder, and/or hazel pollen. A CMA compares the costs per course of treatment under the assumption that the two treatment alternatives have demonstrated equal efficacy. Costs were collected and analysed for two largest provinces in Canada, Ontario and Quebec. Tree Pollen SCIT was identified as the only appropriate comparator for the analysis (Allegro [DIN: 99101142 and 99101107] for the pre-seasonal and annual products specifically, see also Table 2). A CMA was determined to be the most feasible and appropriate type of economic analysis due to the lack of availability of comparative efficacy and safety data for SQ Tree SLIT-tablets against Tree Pollen SCIT. The underlying assumption of therapeutic equivalence could be considered conservative given the evidence supporting a favourable safety profile for SLIT-tablets vs. SCIT [14–16]. Concomitant use of symptom-relieving medicines were assumed to be the same in SLIT-tablets and SCIT patients and have been excluded from the analysis. Nurse costs were not included in the base case analysis, as there was a risk in double counting because these costs are not billed directly through the publicly funded healthcare system. These costs were included as a scenario. A societal perspective was adopted in the base case, including relevant costs of medications, health care services, travel, and productivity losses related to absenteeism, i.e., time off work. The time horizon in the analysis was three years, which corresponds to the minimum treatment course of AIT [12]. A discount rate of 1.5% was applied in accordance with the Canadian Agency for Drugs and Technologies in Health (CADTH) guidelines for the Economic Evaluation of Health Technologies, and reflects common best practices for health economic modelling in Canada [17]. Inputs were sourced from literature and validated by Canadian specialist clinicians (allergists) in active practice in Ontario and in Quebec, respectively.

### Resource use

Three types of resources were considered in the analysis for each product: the immunotherapy treatments themselves, health care resources, and patient resources. Table 1 presents an overview of the base case use of SQ Tree SLIT-tablets and Tree Pollen SCIT during a three-year course of AIT.

**Table 1:** Resource use for SQ Tree SLIT-tablets and Tree Pollen SCIT

Resource	Ontario						Quebec					
	SQ Tree SLIT-tablets			Tree Pollen SCIT			SQ Tree SLIT-tablets			Tree Pollen SCIT		
	Year 1	Year 2	Year 3	Year 1	Year 2	Year 3	Year 1	Year 2	Year 3	Year 1	Year 2	Year 3
10 mL vial (10 inj.) [18]	–	–	–	1^x^	1^x^	1^x^	–	–	–	1^x^	1^x^	1^x^
SQ Tree SLIT-tablets	180	180	180	–	–	–	180	180	180	–	–	–
Number of claims	6	6	6	–	–	–	6	6	6	–	–	–
Start-up visits	1	1	1	–	–	–	1	1	1	–	–	–
GP (5%)*	0.05	0.05	0.05	–	–	–	0.05	0.05	0.05	–	–	–
Specialist (95%)*	0.95	0.95	0.95	–	–	–	0.95	0.95	0.95	–	–	–
Titration visits (1 week between inj.)*	–	–	–	10	10	10	–	–	–	10	10	10
GP (Ontario 10%, Quebec 0%)*	–	–	–	1	1	1	–	–	–	0	0	0
Specialist (Ontario 90%, Quebec 100%)*	–	–	–	9	9	9	–	–	–	10	10	10
Maintenance visits (4 weeks between inj.)*	–	–	–	0	0	0	–	–	–	0	0	0
Follow-up visits	1	1	1	1	1	1	1	1	1	1	1	1
GP (Ontario 10% SLIT, 20% SCIT; Quebec 0%)*	0.1	0.1	0.1	0.2	0.2	0.2	0	0	0	0	0	0
Specialist (Ontario 90% SLIT, 80% SCIT; Quebec 100%)*	0.9	0.9	0.9	0.8	0.8	0.8	1	1	1	1	1	1
Patient’s time [h]*[18]	3^^^	3^^^	3^^^	19.58^#^	19.58^#^	19.58^#^	3^^^	3^^^	3^^^	20.42^#^	20.42^#^	20.42^#^
Patient’s travel distance ([km]; 20 km per visit)*	40	40	40	200	200	200	40	40	40	200	200	200

*GP* general practitioner, *SCIT* subcutaneous immunotherapy, *inj.* Injection, *SQ Tree* SLIT-tablets SQ tree pollen sublingual immunotherapy tablet

^*^Based on physician input, ^x^pre-seasonal: 1 treatment set, ^#^Patient's time include: Travel time round trip 40 min. [18], pre-injection wait time 15 min.*, injection time 5 min [18], post-injection wait time 30 min.*, physician consultation time 20 min.*, ^Patient's time include: Travel time round trip 40 min. [18], wait time 15 min. [18], physician consultation time 20 min. [18], and for year 1 only a 30 min. post-tablet observational time after first tablet intake[18]

For the SQ Tree SLIT-tablets, the recommended dose is one tablet once daily (based on product monographs) initiated prior to the tree pollen season and maintained throughout the season. As tree pollen is a seasonal allergen, it was assumed that the SQ Tree SLIT-tablets would be taken daily for 6 months, which includes both the pre-season and the tree pollen allergy season. For Tree Pollen SCIT, it was assumed that the treatment would be administered on a pre-seasonal basis with 10 weekly injections in the titration phase and no maintenance phase injections would be administered. It was also assumed that one 10 mL vial would last for 10 injections [18]. One treatment set was assumed to be sufficient for an entire pre-seasonal treatment schedule. The same treatment schedule assumptions were made for each year of treatment. The key difference between treatments is the at-home administration of the SQ Tree SLIT-tablets, resulting in lower health care resource use as well as lower patient resource use. In that respect, it was assumed that a SQ Tree SLIT-tablets patient would attend one start-up visit and one follow up visit during each year of analysis. Due to the daily at-home administrations, no further health care resource used was assumed to be associated with SLIT-tablets. It was assumed that Tree Pollen SCIT patients would receive 10 titration injections at a physician’s clinic. A Tree-Pollen SCIT patient was also assumed to attend one follow-up visit per year. These assumptions are conservative as they do not include the health care costs related to adverse events and injection reactions to SCIT. Reactions could result in additional titration visits or, if a reaction is severe (e.g. anaphylaxis), a visit to a hospital emergency department may be necessary, resulting in additional health care resource use and costs.

### Resource costs

The costs of the resources are summarised in Table 2. Assumptions for medication costs were obtained from the manufacturer for SQ Tree SLIT-tablets, provincial formularies/manufacturer submitted price for SCIT, the Ontario Public Drug Programs [19, 20] and Régie de l’assurance maladie du Québec (RAMQ) [21]. Dispensing fees (professional fee for dispensing prescription medications) of $8.83 and $9.28 were applied for each claim of the SQ Tree SLIT-tablets for Ontario and Quebec, respectively [20, 22]. Pharmacy and wholesaler mark-up percentages were not included in this analysis. Costs for the health care services were obtained from the Ontario Schedule of Benefits [23], Manuel des Médécins Omnipracticiens [24], and Living in Canada (Canadian Registered Nurse salary) [25, 26]. Cost estimates for hours of lost work were obtained from Statistics Canada [27], and travel costs per kilometre were obtained from the Government of Canada 2020 Reasonable Allowance Rates [28]. All costs were presented in Canadian Dollars.

**Table 2:** Resource costs for SQ Tree SLIT-tablets and Tree Pollen SCIT

Cost category	Cost type		$CAD/unit	
	Ontario	Quebec	Ontario	Quebec
SQ Tree SLIT-tablets	Box of 30 tablets		132.00	
	Dispensing fee/claim [20, 22]		8.83	9.28
Tree Pollen SCIT vials^*^^#^	Pre-Seasonal Treatment: Presaisonnier- Arbres Complete Treatment [Allegro—99101142] (one treatment) [21]		265.00	
	Annual Treatment—10 mL concentrate—Monovalent standardise [Allegro—99101107] (one vial) [21]			
Physician	Medical specific re-assessment (follow-up visit), specialist consultation, A474 [23]	Initial visit, under 80, In office or at home, less than 500 patients (average)—Code 15801 [24]	63.70	75.13
	Partial assessment (pre- or post-injection), specialist consultation, A478 [23]	Follow-up visit, under 80, In office or at home, less than 500 patients (average)—Code 15803 [24]	38.25	37.55
	Injection (sole reason for visit), G202 [23]	Hyposensitization treatment, without examination, one or more injections in same session—Code 00400 [24]	4.45	5.10
	Injection (with consultation at same visit), G212 [23]		9.75	
Nurse	Hourly wage [25, 26] (Scenario Analysis)		39.22	34.22
Patient	Average hourly wage [27]		28.52	29.01
	Travel expense by private car [28]		0.59	

*SQ Tree* SLIT standardized quality Tree pollen sublingual immunotherapy, *Tree Pollen SCIT* Tree Pollen subcutaneous immunotherapy

*In Canada, a dispensing fee is a professional fee that is charges by the pharmacy for each filled prescription

^#^Physicians noted that pre-seasonal formulations and annual formulations vary but under the Quebec public formulary (RAMQ, [21]), prices for both sets are the same. Other company’s SCIT products were not included in the analyses, however, of note, for the Quebec public formulary, the products from OMEGA are priced similarly, or are more expensive, than the included products

To calculate the costs and potential savings associated with the use of SQ Tree SLIT-tablets vs. Tree Pollen SCIT over the three-year time horizon, the cost per unit of each resource was multiplied by the amount of resource used each year. For Tree Pollen SCIT, the base case assessed the costs and resource use for pre-seasonal treatment while a scenario analysis considered the costs and resource use for annual treatment.

### Scenario analyses

To assess the robustness of the results and the impact of assumptions on the results of the analysis, scenario analyses were performed. Alternative assumptions for parameters such as Tree Pollen SCIT resource use, discount rates, number of injections, annual SCIT dosing with maintenance injections, and nurse time support were considered.

## Results

### Cost of treatment—SQ Tree SLIT-tablets vs. Tree Pollen SCIT

The annual costs of treatment and the results of potential savings associated with the use of three-year treatment with SQ Tree SLIT-tablets vs. Tree Pollen SCIT are summarised in Tables 3 and 4, respectively. The annual cost per treatment for each of the three years are the same for both SQ Tree SLIT-tablets vs. Tree Pollen SCIT: for SQ Tree SLIT-tablets, $1056.09 in Ontario and $1070.99 in Quebec, and for Tree Pollen SCIT, $1628.32 in Ontario and $1648.01 in Quebec (Table 3). The direct costs, including the drug costs, and physician services, for a three-year treatment period were similar for both treatments for both Ontario and Quebec: for SQ Tree SLIT-tablets $2799.01 and $2838.70, respectively, and for Tree Pollen SCIT $2233.76 and $2266.05, respectively (Table 4). The indirect costs, including patient’s travel expenses and hours lost from paid labour, were higher for Tree Pollen SCIT for both Ontario and Quebec: for SQ Tree SLIT-tablets $322.66 and $327.01, respectively, and for Tree Pollen SCIT $1999.70 and $2.099.53, respectively. Overall, the CMA revealed total savings for the treatment with SQ Tree SLIT-tablets of $1111.79 for Ontario and $1199.87 for Quebec over the three-year analysis period (Table 4).

**Table 3:** Costs of SQ Tree SLIT-tablets and Tree Pollen SCIT treatment per year (in $ CAD)

Cost category	Ontario			Quebec		
	Year 1	Year 2	Year 3	Year 1	Year 2	Year 3
SQ Tree SLIT-tablets						
Drug costs	844.98	844.98	844.98	847.68	847.68	847.68
Tablet costs	792.00	792.00	792.00	792.00	792.00	792.00
Dispensing fee	52.98	52.98	52.98	55.68	55.68	55.68
Physician costs	101.95	101.95	101.95	112.68	112.68	112.68
GP costs	8.28	8.28	8.28	1.88	1.88	1.88
Specialists costs	93.67	93.67	93.67	110.80	110.80	110.80
Total Health care costs	946.93	946.93	946.93	960.36	960.36	960.36
Patients costs	109.16	109.16	109.16	110.63	110.63	110.63
Time costs	85.56	85.56	85.56	87.03	87.03	87.03
Travel costs	23.60	23.60	23.60	23.60	23.60	23.60
Total costs	1056.09	1056.09	1056.09	1070.99	1070.99	1070.99
Tree Pollen SCIT						
Drug costs	265.00	265.00	265.00	265.00	265.00	265.00
Physician costs	490.70	490.70	490.70	501.63	501.63	501.63
Injection	44.50	44.50	44.50	51.00	51.00	51.00
GP costs	4.45	4.45	4.45	0.00	0.00	0.00
Specialists costs	40.05	40.05	40.05	51.00	51.00	51.00
Consultation costs	446.20	446.20	446.20	450.63	450.63	450.63
GP costs	50.99	50.99	50.99	0.00	0.00	0.00
Specialists costs	395.21	395.21	395.21	450.63	450.63	450.63
Total Health care costs	951.80	951.80	951.80	937.73	937.73	937.73
Patients costs	676.52	676.52	676.52	710.29	710.29	710.29
Time costs	558.52	558.52	558.52	592.29	592.29	592.29
Travel costs	118.00	118.00	118.00	118.00	118.00	118.00
Total costs	1432.22	1432.22	1432.22	1476.91	1476.91	1476.91

*GP* general practitioner, *SQ Tree* *SLIT standardized quality Tree pollen sublingual immunotherapy*, *Tree Pollen* SCIT Tree Pollen subcutaneous immunotherapy

**Table 4:** Costs and potential savings: three-year treatment SQ Tree SLIT-tablets vs. Tree Pollen SCIT (in $CAD)

Cost category	Ontario			Quebec		
	SQ Tree SLIT-tablets	Tree Pollen SCIT	SQ Tree SLIT-tablets vs. Tree Pollen SCIT	SQ Tree SLIT-tablets	Tree Pollen SCIT	SQ Tree SLIT-tablets vs. Tree Pollen SCIT
Drug costs	2497.66	783.31	1714.35	2505.64	783.31	1722.33
Physician costs	301.35	1450.45	− 1149.10	333.05	1482.75	− 1149.69
Nurse costs	–	–	–	–	–	–
Total health care costs	2799.01	2813.41	− 14.40	2838.70	2771.81	66.89
Indirect costs (patient)	322.66	1999.70	− 1677.04	327.01	2099.53	− 1772.52
Total costs	3121.68	4233.47	− 1111.79	3165.71	4365.58	− 1199.87

*SQ Tree* SLIT standardized quality Tree pollen sublingual immunotherapy, *Tree Pollen* SCIT Tree Pollen subcutaneous immunotherapy

### Scenario analyses

**Fig. 1:**
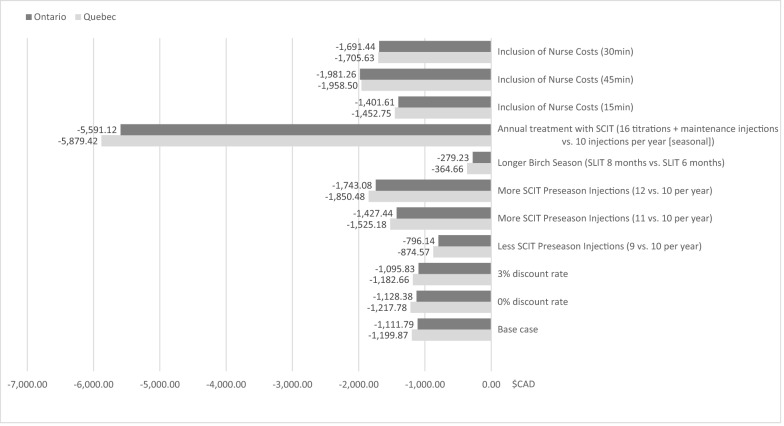
Scenario analyses: potential total savings of SQ Tree SLIT-tablets vs. Tree Pollen SCIT. Potential total savings according to total costs. Total cost of three-year’s treatment are discounted with 1.5% as described in methods, except for the scenario analyses “0% discount rate” and “3% discount rate” where the parameter discount rate was changed to 0 or 3%, respectively. *SQ Tree* SLIT standardized quality birch pollen sublingual immunotherapy, *Tree Pollen* SCIT Tree Pollen subcutaneous immunotherapy

Results of the scenario analyses are shown in Fig. 1. Overall, the scenario analyses demonstrated cost savings with SQ Tree SLIT-tablet treatment compared with Tree Pollen SCIT for each of the scenarios analysed, with some variation in the magnitude of potential savings. Results for the cost difference for treatment over three years with SQ Tree SLIT-tablets compared with Tree Pollen SCIT were sensitive to changes in nurse time per SCIT injection, treatment schedule (annual vs. seasonal for SCIT, longer birch season for SLIT-tablets) and number of SCIT pre-season injections. When the nurse time was included, an estimate of 30 min of nurse time was assumed. The potential savings with SQ Tree SLIT-tablets vs. Tree Pollen SCIT shifted to $1691.44 for Ontario and $1705.63 for Quebec over the three-year analysis period. The most significant cost difference between SQ Tree SLIT-tablets and Tree Pollen SCIT treatment was observed for the scenario of annual treatment with SCIT. Annual treatment is common in clinical practice and the schedule comprises of sixteen weekly titration visits followed by a maintenance visit every four weeks, for the remaining duration of three years of therapy. This analysis resulted in potential savings, for the SLIT-tablets, of $5591.12 for Ontario and $5879.42 for Quebec over the three-year analysis period. The least potential savings were observed for the scenario that assessed a longer birch season (9 vs. 6 months of SLIT-tablets treatment) with projected cost savings for the SLIT-tablets of $279.23 (Ontario) and $364.66 (Quebec). Compared to the base case, almost no impact to costs were observed if the discount rate was changed (from 1.5% to either 0% or 3%) in the scenario analyses.

## Discussion

The current CMA analysed the economic impact of SQ Tree SLIT-tablets compared with Tree Pollen SCIT in Canada for the treatment of tree pollen-induced AR assuming a three year treatment schedule. Overall, the results indicate a cost-minimizing potential of SQ Tree SLIT-tablets for the treatment of tree pollen-induced AR when compared with Tree Pollen SCIT. This result was consistent for all scenarios analysed. When comparing only the direct costs, treatment with SQ Tree SLIT-tablets and Tree Pollen SCIT were similar for both Ontario and Quebec. The higher drug costs for SQ Tree SLIT-tablets vs. Tree Pollen SCIT ($2497.66 vs. $783.31 for Ontario, $2505.64 vs. $783.31 for Quebec) were offset by the healthcare resource costs of physician services, which were more than three times higher for Tree Pollen SCIT. Comparing the indirect costs, these are much higher for Tree Pollen SCIT than SQ Tree SLIT-tablets due to more frequent visits for administrations by health care professionals. Subsequently, when assessing total costs, which resulted in overall potential savings of $1111.79 for Ontario and $1199.87 for Quebec, the CMA indicates that treatment with SQ Tree SLIT-tablets is a cost-minimizing alternative to Tree Pollen SCIT in Canada. This is in line with a recent analysis [29], which reported that in four of six studies comparing cost outcomes of SLIT vs. SCIT, SLIT was the cost-saving therapy.

As Tree Pollen SCIT treatment requires a much higher number of clinic visits, this subsequently results in both higher costs associated with health care professional services and patient resources, thus outweighing the higher drug costs for the SQ Tree SLIT-tablets. With the difference in the number of clinic visits between SQ Tree SLIT-tablets and Tree Pollen SCIT treatment being the main driver of the cost difference, parameters related to the treatment setting had a relatively large impact in the scenario analyses. For example, the most significant savings were obtained in comparison with the annual treatment administration regimen for Tree Pollen-SCIT with the potential of SQ Tree SLIT-tablets to reduce healthcare resource use and associated costs significantly. In addition, at-home administration of SQ Tree SLIT-tablets is more convenient for patients as it decreases the burden of travel and time-off work. At-home administration of SLIT can be particularly advantageous in rural communities, where large distances from the nearest clinic may pose additional barriers to access. With a high number of visits accompanied by a high time load required for SCIT, adherence to treatment could be potentially reduced. Time load of AIT was previously identified as a central factor for patients to ensure optimal adherence to therapy [30]. This is also supported by a patient preference study conducted in Germany using a discrete choice experiment in 239 adults with moderate to severe grass, birch, and/or house dust mite AR. The study found that the attribute most preferred by patients regarding the mode of AIT administration was related to the number and duration of physician visits, with a strong preference for fewer visits with shorter durations [31]. A parallel physician patient survey found that Canadian patients, when asked about their preference for AIT options, were more likely to follow their allergists’ recommendation for initiation of SLIT compared with SCIT [32]. Positive effects on patients’ quality of life have been demonstrated in the pivotal phase III trial for SQ Tree SLIT-tablets during both the birch and tree pollen season [33]. Assessed by the Rhinoconjunctivitis Quality of Life Questionnaire (RQLQ), the SQ tree SLIT-tablets showed a significantly better overall RQLQ than placebo with relative differences of 31% for the birch pollen season and 28% for the tree pollen season (both seasons *P* < 0.0001) [33]. Furthermore, the convenient at-home administration of SLIT-tablets not only frees up health care resources that could be used to help more patients in need of AIT treatment but also reduces public contact events which is favourable in circumstances such as the COVID-19 global pandemic. The current analysis assumes all patients to be 100% adherent to therapy as is customary in analyses like this. Assuming a lower adherence could lead to lower acquisition costs as less medication is dispensed. Adherence to therapy is however an important factor to gain the benefits of treatment. Studies have shown similar compliance rates between SCIT and SLIT over a three-year course of treatment [34–36].

The CMA has some limitations. A CMA builds on an assumption of equal efficacy. Given the lack of head-to-head studies, this assumption is a limitation of this analysis. However, a CMA was determined to be the most feasible and appropriate type of economic analysis, as this assessment method has also been used in similar studies [37–39]. Costs and resource use included in the analysis were solely associated with the treatments and treatment administration, including direct and indirect costs. Other potential aspects related to tree pollen-induced AR were not considered. In addition, certain resource use assumptions were based on input from Canadian allergy specialists in clinical practice in Ontario and Quebec, and there might be geographical and regional variations in resource use in clinical practice. Furthermore, one of areas of uncertainty that exist is the length of the treatment, i.e., whether a 6 month treatment course including 4 month pre-seasonal and 2 month seasonal treatment adequately covers the pollen season. To address this uncertainty an 8 month treatment course with SLIT was included as a scenario analysis, which still demonstrated cost-savings compared to SCIT.

## Conclusions

The CMA estimates the SQ Tree SLIT-tablets to be a cost-minimizing alternative to Tree Pollen SCIT for the treatment of tree pollen induced AR when considered from a societal perspective in both Ontario and Quebec. Scenario analyses which varied resource use, discount rates, number of injections, nurse time, treatment schedule (annual vs. seasonal for SCIT, longer birch season for SLIT-tablets) support this conclusion. All analysed scenarios resulted in savings when treating with SQ Tree SLIT-tablets compared to Tree Pollen SCIT. This CMA demonstrates the cost savings to society associated with introducing SQ Tree SLIT-tablets in Canada.

## Data Availability

The data collected and analysed during the current study are available from the corresponding author on reasonable request.

## Abbreviations

AIT: Allergy immunotherapy
AR: Allergic rhinitis
Bet: Betula (from Betula verrucosa)
CADTH: Canadian Agency for Drugs and Technologies in Health
CMA: Cost minimization analysis
IG: Immunglobulin
LTRA: Leukotriene receptor antagonist
RAMQ: Régie de l’assurance maladie du Québec
SCIT: Subcutaneous immunotherapy
SLIT: Sublingual immunotherapy

## References

[1] Keith PK, Desrosiers M, Laister T, Schellenberg RR, Waserman S (2012). The burden of allergic rhinitis (AR) in Canada: perspectives of physicians and patients. Allergy Asthma Clin Immunol Off J Can Soc Allergy Clin Immunol.

[2] White JF, Bernstein DI (2003). Key pollen allergens in North America. Ann Allergy Asthma Immunol Off Publ Am Coll Allergy Asthma Immunol.

[3] Lok SD, Davis BE, Cockcroft DW (2017). Prevalence of allergen sensitization in 1000 adults in Saskatchewan. Allergy Asthma Clin Immunol.

[4] Ahmed H, Ospina MB, Sideri K, Vliagoftis H (2019). Retrospective analysis of aeroallergen’s sensitization patterns in Edmonton, Canada. Allergy Asthma Clin Immunol Off J Can Soc Allergy Clin Immunol.

[5] Chan-Yeung M, Anthonisen NR, Becklake MR, Bowie D, Sonia Buist A, Dimich-Ward H (2010). Geographical variations in the prevalence of atopic sensitization in six study sites across Canada. Allergy.

[6] Couroux P, Ipsen H, Stage BS, Damkjær JT, Steffensen MA, Salapatek AM (2019). A birch sublingual allergy immunotherapy tablet reduces rhinoconjunctivitis symptoms when exposed to birch and oak and induces IgG4 to allergens from all trees in the birch homologous group. Allergy.

[7] Biedermann T, Winther L, Till SJ, Panzner P, Knulst A, Valovirta E (2019). Birch pollen allergy in Europe. Allergy.

[8] Aerobiology Research Laboratories. Pollen forecasts and collection stations 2020. [Internet]. https://www.pollenexperts.ca/monitoring-network/. Accessed 12 Feb 2020

[9] Small P, Keith PK, Kim H. Allergic rhinitis. Allergy Asthma Clin Immunol Off J Can Soc Allergy Clin Immunol [Internet]. 2018 [cited 2018 Oct 9];14. https://www.ncbi.nlm.nih.gov/pmc/articles/PMC6156899/10.1186/s13223-018-0280-7 PMC6156899 30263033

[10] Wallace DV, Dykewicz MS, Bernstein DI, Blessing-Moore J, Cox L, Khan DA (2008). The diagnosis and management of rhinitis: an updated practice parameter. J Allergy Clin Immunol.

[11] Brożek JL, Bousquet J, Agache I, Agarwal A, Bachert C, Bosnic-Anticevich S (2017). Allergic Rhinitis and its Impact on Asthma (ARIA) guidelines-2016 revision. J Allergy Clin Immunol.

[12] Jutel M, Agache I, Bonini S, Burks AW, Calderon M, Canonica W (2015). International consensus on allergy immunotherapy. J Allergy Clin Immunol.

[13] ALK-Abelló A/S. ITULATEK^TM^ (Standardized Allergen Extract, White Birch (Betula Verrucosa)) product monograph. Hørsholm, Denmark. 2020.

[14] Epstein TG, Liss GM, Murphy-Berendts K, Bernstein DI (2011). Immediate and delayed-onset systemic reactions after subcutaneous immunotherapy injections: ACAAI/AAAAI surveillance study of subcutaneous immunotherapy: year 2. Ann Allergy Asthma Immunol Off Publ Am Coll Allergy Asthma Immunol.

[15] Demoly P, Emminger W, Rehm D, Backer V, Tommerup L, Kleine-Tebbe J (2016). Effective treatment of house dust mite-induced allergic rhinitis with 2 doses of the SQ HDM SLIT-tablet: Results from a randomized, double-blind, placebo-controlled phase III trial. J Allergy Clin Immunol.

[16] Bernstein DI, Wanner M, Borish L, Liss GM (2004). The Immunotherapy Committee of the American Academy of Allergy A and I. Twelve-year survey of fatal reactions to allergen injections and skin testing: 1990–2001. J Allergy Clin Immunol.

[17] Canadian Agency for Drugs and Technologies in Health (CADTH). Guidelines for the economic evaluation of health technologies: Canada (4th edition). 2017;

[18] Blume SW, Yeomans K, Allen-Ramey F, Smith N, Kim H, Lockey RF (2015). Administration and burden of subcutaneous immunotherapy for allergic rhinitis in U.S. and Canadian clinical practice. J Manag Care Spec Pharm.

[19] Ontario Public Drug Programs [Internet]. http://www.health.gov.on.ca/en/public/programs/drugs/. Accessed 06 Oct 2020

[20] PMPRB. (2019). Dispensing fee policies in public drug plans, 2017/18. [Internet]. http://www.pmprb-cepmb.gc.ca/view.asp?ccid=1308. Accessed 06 Oct 2020

[21] Régie de l’assurance maladie du Québec, RAMQ. (Effective September 30, 2020) [Internet]. http://www.ramq.gouv.qc.ca/en/Pages/home.aspx. Accessed 06 Oct 2020

[22] Dispensing fee Quebec—data on file/personal communication.

[23] Ontario Schedule of Benefits (effective April1, 2020) [Internet]. http://www.health.gov.on.ca/en/pro/programs/ohip/sob/physserv/sob_master20200306.pdf. Accessed 09 Sep 2020

[24] Medecins Omnipracticiens (June 2020) [Internet]. https://www.ramq.gouv.qc.ca/SiteCollectionDocuments/professionnels/manuels/syra/medecins-omnipraticiens/100-facturation-omnipraticiens/manuel-omnipraticiens-remuneration-acte-RFP.pdf

[25] 2020 full time average hourly rate (Ontario) [Internet]. https://www.livingin-canada.com/salaries-for-registered-nurses.html. Accessed 06 Oct 2020

[26] 2018 full time average hourly rate (Montreal-Quebec). Adjusted to 2020. [Internet]. https://www.livingin-canada.com/salaries-for-registered-nurses.html. Accessed 06 Oct 2020

[27] Statistics Canada, average hourly wages employees by selected characteristics and occupation [Internet]. http://www.statcan.gc.ca/tables-tableaux/sum-som/l01/cst01/labr69a-eng.htm. Accessed 06 Oct 2020

[28] Government of Canada 2020 Reasonable Allowance Rates [Internet]. https://www.canada.ca/en/revenue-agency/services/tax/businesses/topics/payroll/benefits-allowances/automobile/automobile-motor-vehicle-allowances/reasonable-kilometre-allowance.html. Accessed 06 Oct 2020

[29] Hankin CS, Cox L (2014). Allergy immunotherapy: what is the evidence for cost saving?. Curr Opin Allergy Clin Immunol.

[30] Sondermann N, Shah-Hosseini K, Henkel K, Schwalfenberg A, Mösges R (2011). Erfolgsfaktoren der Adherence bei Hyposensibilisierung. Allergologie.

[31] Damm K, Volk J, Horn A, Allam J-P, Troensegaard-Petersen N, Serup-Hansen N (2016). Patient preferences in allergy immunotherapy (AIT) in Germany - a discrete-choice-experiment. Health Econ Rev.

[32] Ellis AK, Boursiquot J, Carr S, Graham F, Masse M-S. Patient and physician perceptions of seasonal allergic rhinitis and allergen immunotherapy: a parallel physician patient survey. Allergy Asthma Clin Immunol Off J Can Soc Allergy Clin Immunol [Internet]. 2020 [cited 2020 Nov 19];16. https://www.ncbi.nlm.nih.gov/pmc/articles/PMC7035743/10.1186/s13223-020-0412-8 PMC7035743 32123533

[33] Biedermann T, Kuna P, Panzner P, Valovirta E, Andersson M, de Blay F (2019). The SQ tree SLIT-tablet is highly effective and well tolerated: Results from a randomized, double-blind, placebo-controlled phase III trial. J Allergy Clin Immunol.

[34] Allam J-P, Andreasen JN, Mette J, Serup-Hansen N, Wüstenberg EG (2018). Comparison of allergy immunotherapy medication persistence with a sublingual immunotherapy tablet versus subcutaneous immunotherapy in Germany. J Allergy Clin Immunol.

[35] Borg M, Løkke A, Hilberg O (2020). Compliance in subcutaneous and sublingual allergen immunotherapy: a nationwide study. Respir Med.

[36] Hsu NM, Reisacher WR (2012). A comparison of attrition rates in patients undergoing sublingual immunotherapy vs subcutaneous immunotherapy. Int Forum Allergy Rhinol.

[37] Björstad Å, Cardell L-O, Hahn-Pedersen J, Svärd M (2017). A cost-minimisation analysis comparing sublingual immunotherapy to subcutaneous immunotherapy for the treatment of house dust mite allergy in a Swedish setting. Clin Drug Investig.

[38] Ellis AK, Gagnon R, Hammerby E, Lau A (2019). Sublingual immunotherapy tablet for the treatment of house dust mite allergic rhinitis in Canada: an alternative to minimize treatment costs?. Allergy Asthma Clin Immunol Off J Can Soc Allergy Clin Immunol.

[39] Rønborg S, Johnsen C, Theilgaard S, Winther A, Hahn-Pedersen J, Andreasen J (2016). Cost-minimisation analysis of sublingual immunotherapy versus subcutaneous immunotherapy for house dust mite respiratory allergic disease in Denmark. J Med Econ.

